# Dynamic of heat production partitioning in rooster by indirect calorimetry

**DOI:** 10.5713/ab.22.0026

**Published:** 2022-05-02

**Authors:** Rony Lizana Riveros, Rosiane de Sousa Camargos, Marcos Macari, Matheus de Paula Reis, Bruno Balbino Leme, Nilva Kazue Sakomura

**Affiliations:** 1 Faculty of Agricultural and Veterinary Sciences, Sao Paulo State University, Via de Acesso Prof. Paulo Donato Castellane s/n, 14884-900, Jaboticabal, SP, Brazil

**Keywords:** Energy Expenditure, Energy Metabolism, Energy Utilization, Metabolic Rate, Rooster

## Abstract

**Objective:**

The objective of this study was to describe a methodological procedure to quantify the heat production (HP) partitioning in basal metabolism or fasting heat production (FHP), heat production due to physical activity (HPA), and the thermic effect of feeding (TEF) in roosters.

**Methods:**

Eighteen 54-wk-old Hy Line Brown roosters (2.916±0.15 kg) were allocated in an open-circuit chamber of respirometry for O_2_ consumption (VO_2_), CO_2_ production (VCO_2_), and physical activity (PA) measurements, under environmental comfort conditions, following the protocol: adaptation (3 d), *ad libitum* feeding (1 d), and fasting conditions (1 d). The Brouwer equation was used to calculate the HP from VO_2_ and VCO_2_. The plateau-FHP (parameter L) was estimated through the broken line model: HP = U×(R–t)×I+L; I = 1 if t<R or I = 0 if t>R; Where the broken-point (R) was assigned as the time (t) that defined the difference between a short and long fasting period, I is conditional, and U is the decreasing rate after the feed was withdrawn. The HP components description was characterized by three events: *ad libitum* feeding and short and long fasting periods. Linear regression was adjusted between physical activity (PA) and HP to determine the HPA and to estimate the standardized FHP (st-FHP) as the intercept of PA = 0.

**Results:**

The time when plateau-FHP was reached at 11.7 h after withdrawal feed, with a mean value of 386 kJ/kg^0.75^/d, differing in 32 kJ from st-FHP (354 kJ/kg^0.75^/d). The slope of HP per unit of PA was 4.52 kJ/mV. The total HP in roosters partitioned into the st-FHP, termal effect of feeding (TEF), and HPA was 56.6%, 25.7%, and 17.7%, respectively.

**Conclusion:**

The FHP represents the largest fraction of energy expenditure in roosters, followed by the TEF. Furthermore, the PA increased the variation of HP measurements.

## INTRODUCTION

Many factors affect the energy expenditure in poultry. The indirect calorimetry method, together with heat production (HP) measurements, are being used to provide a better explanation of energy metabolism and HP variation factors [1]. A real-time HP measurement can evaluate the animal energy metabolism (and energy utilization) under different conditions and the energy available from the diets or feedstuff. Both components are essential to implementing the net energy (NE) system (requirements and feed energy values) [2].

The HP variations were studied under the effect of different factors. These can be classified as inherent to the animal (e.g., body mass, behavior, physiological state), dependent on the feed characteristics (e.g., Physico-chemical composition, particle size, feed processing, and bio-active components like exogenous enzymes), and environmental factors (e.g., temperature, photoperiod) [2–4]. The HP is partitioned in its components of fasting heat production (FHP) and the thermal effect of feeding (TEF) or heat increment, aiming to elucidate the impact of each factor on energy metabolism. The FHP represents the minimum energy required to sustain life [5]. It is measured when the animal is subject to fasting, under thermoneutrality, and in an inactive circadian phase [6].

On the other hand, the TEF is described as the metabolic heat due to postprandial thermogenesis and metabolic utilization of nutrients, affected principally by the feed chemical composition [1,7,8].

Additionally, another source of energy expenditure is due to physical activity (HPA). In poultry production, the HPA has a low contribution to the total HP; however, it should not be neglected because physical activity (PA) increases the noise of collected data during a continuous measurement of HP. Besides, the physiological response from PA is influenced by metabolic heat expended and energy utilization [9].

This paper describes a procedure to quantify the partitioning of HP in FHP, HPA, and the TEF on roosters.

## MATERIALS AND METHODS

### Animals and management practice

The animal utilization, management and procedures were approved by The Ethics Committee on Animal Use of the Faculdade de Ciências Agrárias e Veterinárias, UNESP, Jaboticabal, São Paulo, Brazil under protocol number n° 013078/19.

Eighteen 54-wk-old Hy Line Brown roosters (2.916±0.15 kg) were used. During the pre-experimental period, the roosters were allocated in individual cages (80×80×75 cm) equipped with feeders and nipple drinkers and maintained at 22°C±2.2°C under a 16 L:8 D light program. The roosters were fed mash type diet (Table 1).

### Experimental protocol

Every 5 d, one bird was randomly selected and transferred to the respiration chamber. The daily management consisted of bird weighting, excreta removal, chamber cleaning, and feed allocation. Daily, the bird's manipulation and chamber saturation lasted 2 h, and this period was not considered for gas exchange calculations.

**Figure 1:**
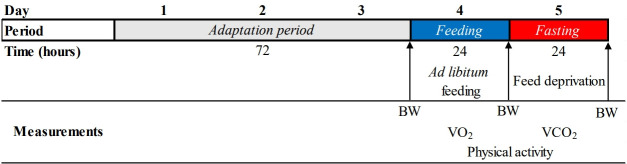
Protocol of bird allocation on the chambers of respirometry and data collection. BW, body weight; VO_2_, oxygen consumption; VCO_2_, carbon dioxide production.

During the data collection, roosters were adapted for 3 d (with free access to feed and freshwater), followed by the measurement of HP under *ad libitum* feeding (~24 h). Then, the feed was withdrawn to measure the HP in the fasting condition (~24 h) (Figure 1).

### Indirect calorimetry and physical activity measurements

**Figure 2:**
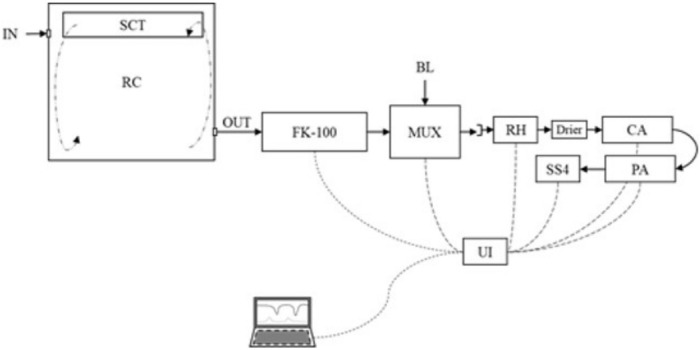
LAVINESP open-circuit calorimetry system. IN, in-going atmospheric air; SCT, temperature controller and air mixing system; RC, cavity for animal allocation; OUT, out-going air from the chamber; FK-100, mass flow pump; MUX, a multiplexer for alternate air sample; RH, water vapor pressure analyzer; Drier, air sample drier; CA, dioxide of carbon analyzer; PA, paramagnetic oxygen analyzer; UI, universal interface data acquisition; SS4, sub-sample air pump. Airflow direction (→). Data transference line (---). Multiplexer was programmed to record 50 minutes for the chamber and 10 minutes for the baseline (This procedure is repeated along a measured period ~24 h).

The gas exchange measurement was done in an open circuit respirometry chamber (dimension: 90×85×95 cm), equipped with environmental temperature control. The respirometry system (Figure 2) consists of a mass flow pump (FK-100; Sable System, Las Vegas, NV, USA) that sucks atmospheric air (by negative pressure) to the inside of the chamber at a flow range between 8 to 12 L/min. The ventilation flow was set to maintain the CO_2_ out-going concentration below 1%. A sub-sampler pump was set at 160 mL/min (SS4; Sable System, USA) to conduct the air sample through the drier (>99.5% CaSO_4_ dihydrate) and gas analyzers. The water vapor pressure was recorded by RH-100 (Sable System, USA), and the O_2_ and CO_2_ were measured using a paramagnetic analyzer (PA-10; Sable System, USA) and infrared analyzer (CA-10; Sable System, USA), respectively. The multiplexer (MUX; Sable System, USA) was programmed to record one data each second, by 3,000 s to chamber gas concentrations recording and 600 s for atmospheric air concentration. This procedure was repeated (in a loop) each hour for 24 h. Average gas concentration every hour was used for HP calculation. The CO_2_ mass recovery factor was 1.032 from a recovery test on the whole respirometry system check.

The physical activity (PA) was measured by an accelerometer (MPU-6050 Three-Axis; MEMS MotionTracking, San Jose, CA, USA) located below the cage to record the vibration (sensibility of ±0.1 mV/s). The cage floor was a solid unfixed platform adapted to transfer the animal's movement with springs on the four vertexes. The PA was recorded each second and averaged every 60 points for further calculations.

### Gas exchange and heat production calculations

According to the Lighton [10] description for an open-circuit system (pull-mode), the gas exchange calculation was done. The in-going airflow (F_in_) to the chamber, oxygen consumption (VO_2_), and production of carbon dioxide (VCO_2_) were calculated according to the following calculations.


(Eq. 1) $${\text{F}}_{\text{in}}\;\left(\frac{\text{L}}{\text{d}}\right)=({\text{F}}_{\text{out}}\times \frac{\left(100-{\left[{\text{O}}_{2}\right]}_{\text{out}}-{\left[{\text{CO}}_{2}\right]}_{\text{out}}\right)}{\left(100-{\left[{\text{O}}_{2}\right]}_{\text{in}}-{\left[{\text{CO}}_{2}\right]}_{\text{in}}\right)}\times 1,440$$

(Eq. 2) $${\text{VO}}_{2}\;\left(\frac{\text{L}}{\text{d}}\right)={\text{F}}_{\text{in}}\times {\left[{\text{O}}_{2}\right]}_{\text{in}}-{\text{F}}_{\text{out}}\times {\left[{\text{O}}_{2}\right]}_{\text{out}}$$

(Eq. 3) $${\text{VCO}}_{2}\;\left(\frac{\text{L}}{\text{d}}\right)={\text{F}}_{\text{out}}\times {\left[{\text{O}}_{2}\right]}_{\text{out}}-{\text{F}}_{\text{in}}\times {\left[{\text{CO}}_{2}\right]}_{\text{in}}$$

Where, F_out_ is the out-going airflow (measured by the FK-100 pump), [O_2_]_in_ and [CO_2_]_in_ is the atmospheric gas concentrations or baseline. VO_2_ and VCO_2_ were calculated from the corrected volume of gas at standard temperature and pressure dry and expressed per unit of metabolic body weight (BW) per day (kg^0.75^/d). The respiratory quotient (RQ) was calculated as $\frac{{\text{VCO}}_{2}}{{\text{VO}}_{2}}$ ratio and the HP were obtained by the Brower equations [11]:


(Eq. 4) $$\text{HP }\left(\frac{\text{kJ}}{{\text{kg}}^{0.75}\text{.d}}\right)=16.18\times {\text{VO}}_{2}+5.02\times {\text{VCO}}_{2}$$

### Heat production description events and partitioning

The HP partitioning was based on metabolic conditions: A, *ad libitum* feeding, where the roosters have free access to feed; B, short fasting period; and C, long fasting period. A segmented model was used to describe the limit between a short and long fasting period, assumed as the variation between the drop of the metabolic cost up to lower stable phases, respectively:


(Eq. 5) HP=U×(R-t)×I+L

Where U is the decline rate of HP (dependent variable) until time R (broken point), L is the plateau value of HP (plateau-FHP), I is the conditional factor (I = 1 if *t*<R or I = 0 if *t*>R), and *t* is the time (independent variable). The parameter R presents the time of transition between a short to a long fasting period (to define the **B** and **C**).

The HP partitioning was described for a rooster (no growth animal) subject to “no limiting” conditions, considering the sum of their components:


(Eq. 6) $${\text{HP}}_{\left(t\right)}=\text{FHP}+{\text{TEF}}_{\left(t\right)}+{\text{HPA}}_{\left(t\right)}$$

The components with (*t*) subindex are assumed to be subject to variation along the measured time.

During event **C**, two components of HP were described without the effect of heat due feeding process, turning feasible to isolate the heat-induced per unit of PA through the linear regression model:


(Eq. 7) $${\text{HP}}_{\left(\text{t}\right)}=\text{FHP}+{\text{HPA}}_{\left(\text{t}\right)}\;\text{or HP}=\beta_{0}+\beta_{1}\times \text{PA}$$

From Eq. 7, the standardized fasting heat production (st-FHP = *β*_0_) and the rate of heat production per unit of PA (HPA = *β*_1_×PA) were estimated.

The TEF_(t)_ was deduced from Eq. 6 and previous st-FHP and HPA_(t)_ calculations (Eq. 7).

### Statistical analyses

VO_2_, VCO_2_, RQ, and HP obtained during the feeding and fasting periods were subjected to one-way analysis of variance analysis. In addition, the HP and time for the segmented model (Eq. 5) were fitted using a non-linear regression procedure. In contrast, linear regression analyses were used for the HP and the PA (Eq. 7). The statistical analyses were performed using the Minitab v.20 statistical software (Minitab Inc., StateCollege, PA, USA).

## RESULTS

**Figure 3:**
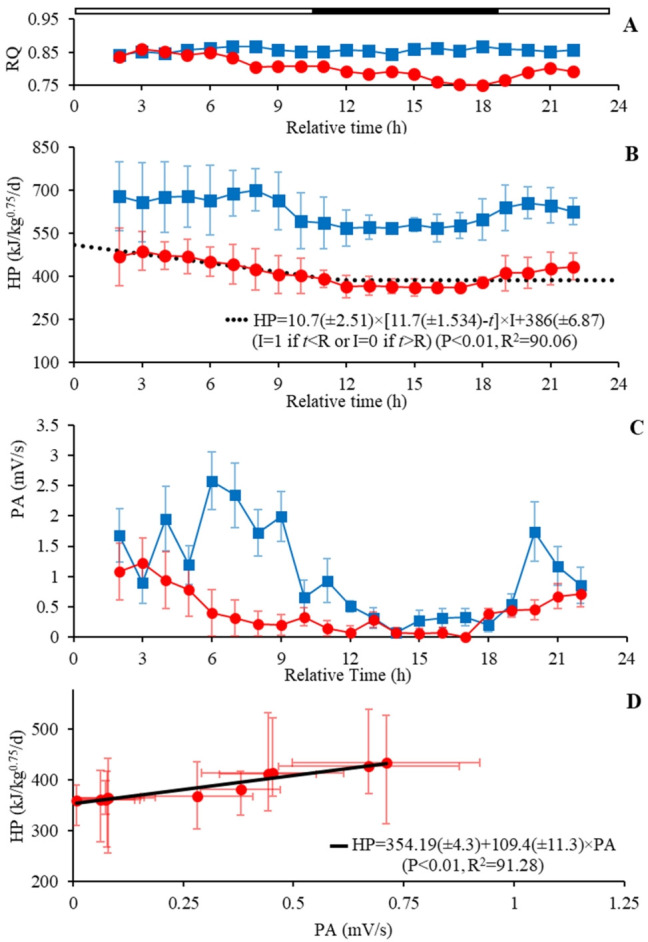
(A) Respiratory quotient (VCO2:VO2), (B) heat production (HP), and (C) physical activity (PA) behavior during the measurement period. The segmented model (•••••) describes the HP decreasing during the fasting period. (D) Regression plot (—) between PA and HP during the long fasting period. The parameters are expressed as value and standard error for the segmented model and linear regression. The top open and close line referred to the light program. R^2^ is the coefficient of determination.

The average values of the gas exchange parameters (VO_2_ and VCO_2_), HP, and RQ are reported for the feeding and fasting period (Table 2). Furthermore, the feed intake (FI) and the metabolizable energy intake (MEI) during the feeding period were reported as the daily average was 0.130±0.017 kg/bird and 720±85 kJ/kg^0.75^, respectively. Roosters under *ad libitum* feeding consumed 14.54% more O_2_ and produced 14.52% more CO_2_ during the light (06 AM to 09 PM) compared to the dark period (10 PM to 05 AM). The triggered reduction of HP was 83 kJ/kg^0.75^/d during the resting (Figure 3B), and a decrease in 65% of PA, from 0.367±0.26 to 1.059±0.77 mV/min of the dark to light period (Figure 3C). On the other hand, the RQ was close during the darkness and light period (0.850 vs 0.856).

The metabolic measurements during the fasting period were low (p<0.01) to VO_2_ (−10.3 L/kg^0.75^/d), VCO_2_ (−9.94 L/kg^0.75^/d), and RQ (−0.055) compared to the *ad libitum* feeding (Figure 3A). Also, the result shows a reduction in metabolic HP of 34.5% for the fasting conditions compared to the feeding period (p<0.01) (Table 2).

The metabolic rate declined after feed deprivation, which significantly fits the segmented model (Figure 3A). The data demonstrate a linear reduction of HP (U = 10.7), starting on the feed withdrawal up to 11.7 h (R) of fasting. After which, the HP reached the plateau-FHP (386.37 kJ/kg^0.75^/d). The RQ associated with the plateau-FHP obtained was 0.751±0.018.

**Figure 4:**
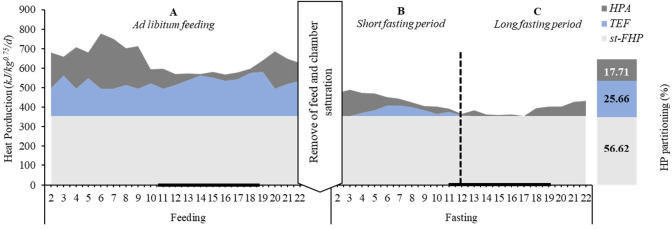
Scheme form the average of all data (n = 18) of heat production partitioning on sequentially period of continuous feed available (A), around 12 h after feed deprivation (B) and long-time of fasting between 11.7 to 24 h of feed deprivation (C). **A** and **B** limits are estimated from the broken-line model (---).

Based on the average of HP reported along the measurement periods investigated herein (Table 2; Figure 3), the events (A, *ad libitum* feeding; B, short fasting; and C, long fasting periods) and HP components are illustrated in Figure 4. When the roosters had free access to the feed (A), all HP components varied along the day. These variations were affected by the lighting and feeding process at each time. Sequentially, the roosters under a short fasting period (B) demonstrate a reduction in HP, where TEF was most affected by feed restriction and a lesser extent, by PA reduction. In a long fasting period (C), the HP has been constituted by the st-FHP and HPA. The linear regression model estimated the PA effect on total HP (p<0.01) between energy expenditure per unit of movement at 4.52 kJ/mV (*β*_1_), and the st-FHP was calculated as the intercept (*β*_0_ = 354 kJ/kg^0.75^/d) (Figure 3).

Two different values that describe the basal metabolic rate could be contrasted. The plateau-FHP was estimated from the broken-line model on the plateau phases after a long fasting period. Moreover, the st-FHP was estimated, considering the PA and isolating their effects. The st-FHP was lower (−42 kJ/kg^0.75^/d) than plateau-FHP.

With the result of previous calculations, was calculated the values of metabolizable energy (ME) partitioning in heat increment (described for this proposed as TEF+HPA, 275 kJ/kg^0.75^/d), 386 kJ/kg^0.75^/d of NEm (or st-FHP), and 59 kJ/kg^0.75^ of retained energy (NEp). Also, the energy efficiency of utilization was $61.85\%\;(\text{NE}=\frac{\left({\text{NE}}_{\text{m}}+{\text{NE}}_{\text{p}}\right)}{\text{ME}})$.

On average, the energy cost observed for a rooster is described as 56.6%, 25.7%, and 17.7% to st-FHP (386 kJ/kg^0.75^/d), TEF (159 kJ/kg^0.75^/d), and HPA (116 kJ/kg^0.75^/d), respectively (Figure 4).

## DISCUSSION

Describing the partition of HP in roosters could be helpful to understand better the feed energy utilization and establish the bird's requirements [12–14]. Therefore, this study shows the energy utilization of roosters to describe better HP partitioning and determine the maintenance requirement.

Indirect calorimetry parameters in laying breed roosters are rarely reported. A pioneering study in fed male broiler breeders reports similar results for gas exchange parameters than we found, 33.85 of O_2_ consumption and 34.87 CO_2_ productions (in L/kg^0.75^/d) [15]. In contrast, Barnas et al [16] conducted a study on laying cockerels reporting similarities with our findings and a slight reduction compared with meat-type breeders for VO_2_ (32 L/kg^0.75^/d) and VCO_2_ (27.68 L/kg^0.75^/d). Otherwise, a wide variation on the RQ is reported in the literature for poultry under fed conditions. In this sense, lower values of 0.86 were reported for laying breeders [16] and higher values close to 1.03 [15] in male broiler breeders. Conceptually the RQ is related to the oxidation rate depending on the substrate [17]. Also, in *ad libitum* fed animals, the RQ is close to 1 [1].

Additionally, the amount and frequency of feeding influenced this value [18]. In this way, broiler breeders present high FI (around 0.163 kg/bird) and a higher frequency of feeding as reported by Fuller et al [15], inducing heavy-fatty chickens, higher compared with male laying breeders [19] and besides higher values of RQ. Also, O'Neil et al [4] describe a wide variation in the RQ in fed roosters with different levels of a single diet and the same BW (around 2.5), varying from 0.814 to 1.050. In recent studies conducted by Liu et al [20] in broiler breeders to evaluate different diets ranging in their chemical composition with the same ME (12.96 MJ of ME/kg) and same FI (0.239 kg/bird), the authors observed no difference in the RQ (average of 0.96). The RQ variation is more related to the amount of FI (affected by the circadian rhythm) and the strain of the chickens (differentiated by the body composition).

Even with these variations in the RQ, Fuller et al [15] and Barnas et al [16] reported the same daily HP per unit of metabolic BW compared with our results, between 661 and 698 kJ/kg^0.75^.

In the continuous measurement of HP throughout the day, many random factors are a source of variation, like crowing [21], spontaneous PA [22], behavior states [23], and principally by the circadian cycle regulation and photoperiod [24]. Since HP is a dynamic phenomenon that constantly changes per unit of time, the calculations in short-time trials can result in noisy data [1]. The variation in HP is governed primarily by the locomotion activity induced by the light program [24]. Gleeson et al [9] reported relative reduction for the gas exchange parameters during the resting, beginning 0.3 and 0.92 L/kg^0.75^/d lower for VO_2_ and VCO_2_, respectively. That produced a numerical (non-significant) reduction in the HP with around nine kJ/kg^0.75^/d of difference between light and dark. Also, no difference was shown for the RQ, with an average reported of 0.930. On the other hand, Caldas et al [13] showed that female broiler breeders presented a significant variation in the gas exchange parameters affected by the light program adopted, reporting reductions of 27% (from 23 to 16.9 L/kg^0.75^/d) and 30% (from 21.9 to 15.4 L/kg^0.75^/d) for VO_2_ and VCO_2_, respectively.

We observed intermediate values than Gleeson et al [9] and Caldas et al [13], keeping a reduction of 15% during the dark period for both gases. Also, we showed a decrease of 65% in the locomotion of the roosters during the resting period.

Energy utilization (NE/ME) efficiency was reported at around 63% [25] for cockerels was closer than we found. The energy utilization in roosters is explained as the nutrient and energy intake destined mainly for maintenance (around 53.61% of ME). Also, a small fraction will be retained as lipid or turnover tissue (NEp around 8.24% of ME), making energy use by mature and non-productive animals less efficient.

The FHP determination depends on the methods used to estimate HP extrapolation at zero MEI [20] or submit the animals for a long time of fasting [8]. Also, the HP measurement under fasting conditions is broadly accepted and used for farm animal trials. Still, it presents some limitations, like guaranteeing the resting state after a long-time feed deprivation [6,26]. Some studies used the minimum HP plateau value to express basal metabolism [27]. Nonetheless, the observation from the present investigation demonstrates a necessity to isolate the effect of FHP for zero PA. In this sense, PA control should be essential to measure and separate the HPA contribution from FHP. The evaluation of metabolic trail conditions, like chamber size, light program, and animal behavior, must be carefully accounted for and monitored to reduce the roosters' displacement inside the chamber. Considering the experimental conditions, PA variation is expected to decline, allowing a better plateau-FHP estimation, matching with the basal metabolic rate of roosters.

As explained, a simple way to estimate the FHP relies on identifying a low asymptote or plateau in the HP after feed deprivation [23]. Still, it needs mechanisms or conditions to guarantee the animal’s inactivity as much as possible [22]. Nevertheless, it is essential to know how much time the roosters need to be fasting to express the plateau-FHP. Noblet et al [8] mentioned that six hours after the last meal plus eight hours of feed deprivation is insufficient for young chickens to reach the plateau of FHP. Furthermore, Zubair and Leeson [27] obtained a constant minimum value of HP for 17-d-old chicks submitted to fasting between 24 to 36 h. Similar results were reported by Liu et al [18]. The latter found a constant RQ (0.65 to 0.75) between 12 and 36 h of fasting for broiler chickens. In our study, a plateau in the HP was observed after 11 h of fasting, therefore reaching the plateau-FHP; however, a longer time (24 h) of fasting might be necessary to achieve a reliable estimate of plateau-FHP [8].

As observed in this study, the plateau-FHP value was higher than st-FHP because even in fasting conditions, the PA effect increased the plateau-FHP estimation. Thus, they denote the importance of accounting for the bird's PA when estimating the basal metabolic rate.

The st-FHP obtained herein was close to that obtained by Rabello et al [25] (360 kJ/kg^0.75^/d) in broiler breeders, O'Neill et al [4] (308 kJ.kg^0.75^/d) in cockerels, and Johnson and Farrell [28] (359 kJ/kg^0.75^/d) discounting the PA effect. Thus, the slight difference between FHP found in literature is probably due to genetic variation and the methodologies applied to estimate the HP [1].

The TEF has low variation compared with the HPA, and it is mainly affected by the frequency of feed consumption and feed behavior, influenced by the light program when the bird has free access to feed [24].

The HPA fraction has more variation, which causes more noise in the trials of HP measurements. Still, it can be stimulated by the feeding period, light program, and other disturbances like crowing due to normal rooster behavior [21]. This fraction is more expressed during the feeding period. Still, it is not feasible to isolate this effect because it is combined with the feeding and the metabolic cost involved in the ingestion and digestion process.

The HP partitioning result was similar to van Milgen et al [26] reports on broiler chicks and Rivera-Torres et al [29] on growing turkeys. These studies partitioned the HP into three components, including heat increment related to the thermic effect of feeding (about 17% to 31% of total HP), FHP (around 52% to 29%), and HPA (17% to 23%), respectively.

In conclusion, the heat production partitioning into the FHP, TEF, and considering the HPA effect for each component improve the interpretation data of energy utilization in laying roosters. The FHP represents the major fraction of energy cost, followed by the TEF depending on the quantity of FI and chemical characteristics. Also, PA has a considerable impact on energy expenditure measurements. Therefore, more accurate maintenance regimes could be developed by PA measurement and quantification and their corresponding energy cost.

## Figures and Tables

**Table 1:** Diet and nutritional composition

Item	%
Ingredients	
Corn, grain 7.86% CP	69.70
Soybean meal 45% CP	25.54
Wheat bran - Midds	1.54
Limestone	1.25
Dicalcium phosph.	1.50
Salt	0.25
DL-Methionine	0.02
Vitamin and mineral premix^1)^	0.20
Total	100
Calculated nutrient composition	
ME (MJ/kg)	12.33
Crude protein	17.32
Total calcium	0.942
Available phosphorus	0.375
Lysine SID	0.777
Methionine SID	0.274
Ether extract	4.352
Starch	43.99
Crude fiber	2.892

CP, crude protein; ME, metabolizable energy; SID, standardized ileal digestibility.

1) Provided the following per kilogram of diet: vitamin A, 8,800 IU; vitamin D3, 3,300 IU; vitamin E, 40 IU; vitamin K_3_, 3.3 mg; thiamine, 4.0 mg; riboflavin, 8.0 mg; pantothenic acid, 15.0 mg; niacin, 50 mg; pyridoxine,3.3 mg; choline, 600 mg; folic acid, 1.0 mg; biotin, 220 g; vitamin B_12_, 12 g; ethoxyquin, 120 mg; Mn, 70 mg;Zn, 70 mg; Cu, 10 mg; Fe, 60 mg; I, 1.0 mg; and Se, 0.3 mg.

**Table 2:** Average gas exchange parameters of oxygen consumption (VO_2_) and carbon dioxide production (VCO_2_), calculations of heat production (HP), and respiratory quotient (RQ)

State		VO_2_ (L/kg^0.75^/d)	VCO_2_ (L/kg^0.75^/d)	RQ	HP (kJ/kg^0.75^/d)
Feeding	μ	30.7	26.3	0.856	628
	SD	6.12	5.42	0.056	125
Light	μ	32.3	27.6	0.856	661
	SD	6.66	6.03	0.058	137
Dark	μ	28.2	24.1	0.850	578
	SD	4.07	3.28	0.051	81
Fasting^1)^	μ	20.4	16.3	0.801	412
	SD	3.56	2.94	0.015	72
SEM		0.790	0.410	0.0001	305
P^2)^		<0.001	<0.001	<0.001	<0.001

SEM, standard error of the mean.

1) Calculated as an average of heat production before the feed was withdrawn along with ~24 h of fasting.

2) Probability reported from analysis of variance analysis between Feeding versus Fasting. The results were expressed as the average (μ) and the standard deviation (SD).
